# Socioeconomic Factors Associated with Household Food Insecurity in Guamote Canton, Ecuador

**DOI:** 10.3390/nu18132110

**Published:** 2026-06-28

**Authors:** Víctor Dante Ayaviri-Nina, Jhonnatan Geovanny Paltan Guzmán, Yaira Brigithe Tello Vallejo, Gabith Miriam Quispe Fernández, Ariana Saraiva, Hmidan A. Alturki, Fahad Alofan, Esteban Pérez-García, António Raposo

**Affiliations:** 1 Facultad de Ciencias Políticas y Administrativas, Universidad Nacional de Chimborazo (UNACH), Riobamba 060103, Ecuador; dayaviri@unach.edu.ec (V.D.A.-N.); jhonnatan.paltan@unach.edu.ec (J.G.P.G.); yaira.tello@unach.edu.ec (Y.B.T.V.); gquispe@unach.edu.ec (G.M.Q.F.); 2 Research in Veterinary Medicine (I-MVET), Faculty of Veterinary Medicine, Lisbon University Centre, Lusófona University, Campo Grande 376, 1749-024 Lisboa, Portugal; ariana.saraiva@ulusofona.pt; 3 Veterinary and Animal Research Centre (CECAV), Faculty of Veterinary Medicine, Lisbon University Centre, Lusófona University, Campo Grande 376, 1749-024 Lisboa, Portugal; 4 King Abdulaziz City for Science & Technology, Wellness and Preventive Medicine Institute—Health Sector, Riyadh 11442, Saudi Arabia; halturki@kacst.edu.sa; 5 Department of Business Administration, College of Business & Economics, Qassim University, Buraydah 51431, Saudi Arabia; f.alofan@qu.edu.sa; 6 Department of Animal Pathology and Production, Bromatology and Food Technology, Faculty of Veterinary, Universidad de Las Palmas de Gran Canaria, Trasmontaña s/n, 35413 Arucas, Spain; esteban.perezgarcia@ulpgc.es; 7 CBIOS (Research Center for Biosciences and Health Technologies), ECTS (School of Health Sciences and Technologies), Lusófona University, Campo Grande 376, 1749-024 Lisboa, Portugal

**Keywords:** educational level, food insecurity, Guamote Canton, household income, public health nutrition, socioeconomic factors, vulnerable populations

## Abstract

Background/Objectives: Food insecurity remains a major public health challenge in Guamote Canton, Ecuador, particularly in areas characterized by high levels of poverty and limited educational attainment. This study examined the association between household income, educational level, and food insecurity in Guamote Canton. Methods: A cross-sectional survey based on the Latin American and Caribbean Food Security Scale (ELCSA) was administered to 368 households, allowing food insecurity to be classified into four categories: food security, mild insecurity, moderate insecurity, and severe insecurity. An Ordinal Logistic model was applied to identify the socioeconomic factors associated with food insecurity. Results: The results showed that household income and educational level were significantly associated with lower probabilities of food insecurity (β = −0.639, *p* = 0.001; β = −0.366, *p* = 0.008, respectively). Gender, Area of residence, and Access to nutrition information were also significantly associated with food insecurity. Conclusions: Households located in rural areas and those headed by women were more likely to experience moderate or severe food insecurity. Overall, the findings highlight the importance of improving income opportunities, educational attainment, and structural living conditions, which may contribute to lower levels of food insecurity.

## 1. Introduction

Food insecurity represents a global challenge that affects a significant proportion of the world’s population and remains a major concern in both developed and developing countries [1,2,3,4]. The concept encompasses constraints in food availability, access, utilization, and stability, which affect the nutritional status and well-being of households [5,6]. Current evidence indicates that food insecurity is associated with a wide range of socioeconomic factors, making it a multidimensional phenomenon that requires ongoing analysis from different perspectives [7,8].

Among the factors most frequently examined in the literature, income and educational attainment have received considerable attention [9,10,11,12]. Income has been associated with households’ ability to access adequate and nutritious food, while educational attainment has been linked to employment opportunities, access to information, and knowledge related to nutrition and resource management [13,14,15,16,17,18,19,20]. Consequently, both variables have been widely incorporated into studies seeking to understand differences in food security conditions among populations.

Empirical evidence from different regions supports the association between income, education, and food insecurity. Studies conducted in Asia have reported that households with lower income levels and limited educational opportunities tend to experience higher levels of food insecurity [21,22,23,24]. Similarly, income diversification and educational attainment have been identified as relevant factors in the analysis of food security conditions and household vulnerability [25,26,27,28,29,30]. Research focused on rural areas has also highlighted the relevance of these variables, particularly in contexts characterized by limited access to markets, services, and employment opportunities [22,31,32,33,34,35].

In Latin America, the relationship between socioeconomic conditions and food insecurity has been extensively documented. Low-income levels and low educational attainment have been associated with a higher likelihood of food insecurity among households [20,31,36,37]. Similar findings have been reported in Ecuador, particularly in rural areas where economic activities depend heavily on agriculture and where access to education and basic services remains unequal [38,39]. Previous studies conducted in Ecuador have also identified education and household income as relevant factors in the analysis of food security conditions [40,41,42,43].

Food insecurity has been described as a multidimensional phenomenon that disproportionately affects rural and low-income populations [44,45,46,47]. Within this framework, economic and educational variables have been systematically incorporated into empirical analyses due to their association with household access to food and conditions of vulnerability [48,49].

The Canton of Guamote represents a particularly relevant case for the study of food insecurity. According to official statistics, the canton has high levels of poverty and educational limitations, while the majority of its population belongs to indigenous communities [38,50]. These characteristics place Guamote among the most vulnerable territories in Ecuador in terms of food security conditions.

Although the literature has extensively examined the relationship between income, education, and food insecurity in international, Latin American, and Ecuadorian contexts [13,14,15,16,17,18,19,20,21,22,23,24,25,26,27,28,29,30,31,36,37], there is little empirical evidence available for highly vulnerable rural indigenous territories such as the Canton of Guamote. Therefore, the objective of this study is to analyze the relationship between income and educational level and food insecurity in the Canton of Guamote, Ecuador. The research is guided by the following question: How are income and educational levels associated with food insecurity in the Canton of Guamote? Based on the evidence reported in the literature, the working hypothesis proposes that income and educational level are negatively associated with food insecurity.

## 2. Methodological Aspects

The study adopts a hypothetical-deductive approach, which allowed the formulation of hypotheses based on existing theory and the deduction of conclusions through the study of empirical data. The type of research corresponds to a descriptive study. It is descriptive because it focuses on characterizing the phenomenon of food insecurity by analyzing its current conditions. The research design corresponds to a non-experimental study, given that the study variables are not manipulated.

The study population consisted of households in the three parishes of the Guamote Canton (Matriz, Cebadas, and Palmira), with a total of approximately 8742 households. Using proportional probability sampling, a sample of 368 surveys was determined, since the Guamote Canton comprises three parishes (Matriz, Cebadas, and Palmira), and given that there are significant differences among them, the application of the proportional sampling method is necessary. This method allows for the determination of the number of surveys to be administered in each parish based on its proportion of the total population, as shown in Table 1.

The inclusion criteria considered individuals over 18 years of age who were permanent residents of the Guamote Canton and responsible for providing information on the household’s socioeconomic and dietary conditions. Incomplete surveys, households consisting of individuals under 18 years of age, and participants who did not agree to take part in the study were excluded.

Data collection was conducted through in-person surveys administered directly in households during the period from August 2024 to October 2024. Interviewers received prior training on the research objectives, the questionnaire content, and the administration procedures to ensure the quality and consistency of the information collected.

The questionnaire was administered in Spanish. Before the survey was administered, the purpose of the research was explained to the participants, and their informed consent was obtained voluntarily. The information collected was treated confidentially and used exclusively for academic purposes. The research was conducted at the National University of Chimborazo, in accordance with the research ethics manual, and was approved by the Ethics Committee of the National University of Chimborazo (UNACH) on 26 July 2024, with the following approval code: 110-DFCPAA-UNACH.

### ELCSA Questionnaire and Validation

The information was collected through the application of a questionnaire based on the Latin American and Caribbean Food Security Scale (ELCSA), supplemented with questions about income and educational level. To verify the reliability of the instrument, a pilot survey was applied that allowed the calculation of Cronbach’s Alpha coefficient [51,52,53]. The value obtained was 0.7998, which showed that the questionnaire presents acceptable internal consistency.

The dependent variable is food insecurity. Since the sampled households in this study consisted of adults (with no members under 18 years of age), the 8-item version of the ELCSA was applied (the complete list of items is provided in Appendix A).

Household classification was determined by the sum of affirmative responses, adhering to the validated thresholds established by the ELCSA guidelines for adult-only households: food security ([0 encoding], 0 affirmative responses), mild food insecurity ([1] 1 to 3), moderate food insecurity ([2] 4 to 6), and severe food insecurity ([3], 7 to 8) [54].

The main independent variables are the economic income and educational level of the head of household, as well as control variables such as gender, area, household size, ethnicity, and type of residence, which were taken into account according to the existing literature.

## 3. Ordinal Logistic Regression Model

For model estimation, both the Ordinal Logit and Probit models were considered, since these models are appropriate for estimating the probability that a household will be located at one of the food insecurity levels based on its income and educational level [17,22]. Model selection was made based on the following statistical criteria: Chi-square test, Pseudo R^2^, maximum likelihood, and the AIC and BIC information criteria, which ensure the validity and robustness of the model [55,56,57].

The model equation is presented below and followed in Table 2 of Coding the Independent Variables:

Pr(Y ≤ j) = 1/{1 + exp(−αj + β_1_IEi + β_2_OIi + β_3_Edui + β_4_Geni + β_5_Zoni + β_6_THi + β_7_AIi + β_8_Etni + β_9_Resi + εi)}

where

Y = dependent variable (food insecurity);
j = represents the different categories of food insecurity: Food security (0), Mild food insecurity (1), Moderate food insecurity (2), and Severe food insecurity (3);
αj, β_1_, β_2_, β_3_, β_4_, β_5_, β_6_, β_7_, β_8_, β_9_ = parameters to be estimated in the regression;
αj = model constant (intercept);
β_1_ = coefficient associated with IEi;
β_2_ = coefficient associated with OIi;
β_3_ = coefficient associated with Edui;
β_4_ = coefficient associated with Geni;
β_5_ = coefficient associated with Zoni;
β_6_ = coefficient associated with THi;
β_7_ = coefficient associated with AIi;
β_8_ = coefficient associated with Etni;
β_9_ = coefficient associated with Resi;
IEi = Economic Income (monthly household income in $);
OIi = Other income: vouchers, internal remittances, and international remittances (in $);
Edui = Education (educational level of the head of household);
Geni = Gender of the person surveyed (male or female);
Zoni = Location of residence, area: urban or rural;
THi = Household size (number of people living in the household);
AIi = Access to information on adequate nutrition;
Etni = Ethnicity of the person surveyed;
Resi = Type of residence (owned, rented, provided by relatives/friends, in antichresis, or other);
εi = errors or residuals.

## 4. Results

The descriptive statistics of the main study variables are presented below:

### 4.1. Food Insecurity Levels in Guamote Canton

In the present research, the dependent variable is food insecurity, which is presented at the following levels: food security, mild food insecurity, moderate food insecurity, and severe food insecurity [58].

A total of 36.41% of households present mild food insecurity, followed by 35.05% with moderate insecurity and 18.75% with severe insecurity; only 9.78% report food security. This suggests that the vast majority of households face some level of food insecurity.

### 4.2. Socioeconomic Characteristics of Households of the Guamote Canton Population

#### 4.2.1. Monthly Household Income

One of the explanatory variables is the monthly household income; at higher income levels, households have more economic resources to purchase nutritious high-quality food, which helps reduce food insecurity [59,60,61].

Nearly 96% of households report incomes below $920. Only 3.54% exceed this amount, evidencing a high concentration of the sample at low-income levels, reflecting the economic limitations faced by these families.

#### 4.2.2. Education Level

The educational level of the head of household is a relevant explanatory variable, as a high education level is linked to higher income. This, in turn, is associated with lower levels of food insecurity, given households’ access to the necessary resources for meeting their basic needs.

44.57% of heads of household have completed secondary education, 27.7% primary education, 17.12% higher education, and 1.6% postgraduate studies. 9.0% have no formal education at all. These results evidence a predominant educational level at the basic and secondary levels among heads of household in the Guamote Canton.

#### 4.2.3. Other Income

Regarding the economic income of households in the Guamote Canton, additional income obtained is also considered, such as government assistance or transfers, both domestic and international, from family members. These complementary incomes play an important role in the fight against food insecurity, providing additional resources that meet households’ basic needs [62].

75.27% of households do not receive additional income. Among those who do receive other income, government vouchers represent 23.64%, while domestic or international remittances barely reach 0.54% of the sample.

#### 4.2.4. Control Variables

Regarding the other control variables: in terms of gender distribution, 70.11% of heads of household are men and 29.89% are women, evidencing a male predominance. Regarding household size, the majority of families (51.63%) have 4 to 7 members, followed by small households (46.20%, with 1 to 3 members) and a small proportion (2.17%) with 8 or more members.

The surveyed population is predominantly indigenous (85.05%), followed by mestizo (14.40%) with minimal presence of white population (0.54%), with no representation of other ethnic groups. Regarding housing, 63.59% is owned, 28.26% is rented, and 8.15% is provided by relatives or friends.

Regarding Access to nutrition information, 52.72% of households do not have access to this resource, while 47.28% do. Finally, 54.08% of the sample resides in urban areas and 45.92% in rural areas, reflecting a relatively balanced distribution between both areas.

### 4.3. Econometric Estimation of the Association Between Household Income, Education Level of the Head of Household, and Food Insecurity in the Guamote Canton

#### 4.3.1. Model Selection

For model estimation, both the Ordinal Logit and Probit models were considered; regressions were carried out with both models for comparison of variable coefficients (see Table 3).

#### 4.3.2. Model Selection Criteria

##### Chi-Square Test

The Chi-square test evaluates whether the independent variables, considered jointly, are significant for explaining the dependent variable in the model. Its null hypothesis states that the variables together do not have a significant effect, which would imply that the model does not have statistically significant predictive capacity for the dependent variable. The objective is to reject the null hypothesis. To do so, it is verified whether the probability associated with the Chi-square statistic is low compared to the established significance level (5%).

In this case, for both the Ordinal Logit and Ordinal Probit models, it is observed that the *p*-value is less than 5%. This allows the rejection of the null hypothesis in both models. However, the Logit model had a slightly higher chi-square value, reflecting greater relative explanatory power compared to the Probit model.

##### Pseudo R^2^

Pseudo R^2^ is a metric used in non-linear models; it indicates how well the model fits the observed data. It is important to note that the value in the table does not reflect the exact percentage of explained variability, but rather a relative measure that allows comparison of models within the same analysis.

In the results obtained, the Ordinal Logit model shows a Pseudo R^2^ of 0.0654, while the Ordinal Probit model has a value of 0.0635. This suggests that the Ordinal Logit model achieves a slightly better fit than the Ordinal Probit model in terms of its capacity to explain the data. Although the difference between the two values is small, the Ordinal Logit presents a relative advantage in this respect.

##### Maximum Likelihood

Maximum likelihood represents the maximum value reached by the likelihood function (log probability of the data) when fitting the proposed models. This value allows the evaluation of how well a model adapts to the observed data.

Table 4 shows that the Ordinal Logit model has a maximum likelihood value of −439.06898, while the Ordinal Probit model reaches a value of −439.96299. Although both models present similar values, the Ordinal Logit model has a higher maximum likelihood value (less negative). This indicates that the Ordinal Logit model provides an optimal fit to the observed data compared to the Ordinal Probit model.

##### Information Criteria

AIC and BIC are statistical criteria used to evaluate and compare models, taking into account both their capacity to fit the data and the associated complexity.

Regarding AIC, the Ordinal Logit model shows a lower value (902.138) than the Ordinal Probit model (903.926), indicating that the Ordinal Logit model achieves a good balance between fit and simplicity. Similarly, the BIC of the Ordinal Logit model (949.035) is lower compared to that of the Ordinal Probit model (950.823), reinforcing the idea that the Ordinal Logit is more efficient and parsimonious according to this criterion. Therefore, when the lowest values are shown in both criteria, it is concluded that the Ordinal Logit model is preferable to the Ordinal Probit in this analysis. This suggests that the Ordinal Logit model offers a better balance between model fit and parsimony.

Despite the similarity between both models, the Chi-square test, the Pseudo R^2^, the maximum likelihood value, together with the AIC and BIC criteria, reinforce the choice of the Ordinal Logit model as the most appropriate for representing the data in this analysis, as it offers a slightly good balance between fit and simplicity.

On the other hand, it is fundamental to consider the literature related to the use of the Ordinal Logit model for food insecurity analysis. In this regard, there are studies that have used this model; for example: in one study, an Ordinal Logit model was used to analyze and evaluate the factors influencing food insecurity in rural households in the Paute River Basin, Azuay province, Ecuador [40]. Similarly, an Ordinal Logit model was used to identify and evaluate how different factors are associated with the probability that households experience different levels of food insecurity, including demographic characteristics, mental health status, and the presence of patients with chronic conditions in households [63]. Finally, the model is used to address food insecurity among beneficiaries of the Popular Dining Program in Mexico City [64]. Through the analysis of beneficiary satisfaction, they evaluate how food quality and other factors relate to food insecurity in households that use this service. In this way, there is empirical evidence of the use of the Ordinal Logit model for the study of food insecurity. The model equation for the present research is presented below.

#### 4.3.3. Multicollinearity Assessment

To assess potential multicollinearity among the explanatory variables, Pearson correlation coefficients were calculated. Table 5 presents the correlation matrix of the independent variables.

#### 4.3.4. Ordinal Logistic Regression Results

The following results were obtained in the first regression conducted (Table 6):

The results show that the variables monthly household income, education level of the head of household, gender, area, and access to information on adequate nutrition are significant at 5%. This means that these variables are significantly associated with the probability that a household belongs to a given food insecurity category.

The table also shows the odds ratios, which indicate that monthly household income and educational level are significantly associated with a lower probability of experiencing higher levels of food insecurity. In contrast, female-headed households and those with information on healthy eating were more likely to fall into higher categories of food insecurity. Likewise, rural households were more likely to experience food insecurity compared to urban households. The other variables included in the model were not statistically significant. Furthermore, the 95% confidence intervals allow for an assessment of the precision of the estimates. An interval that does not include the value 1 indicates a statistically significant association. The Variance Inflation Factor, with an average value of 1.19, indicates the absence of multicollinearity issues in the estimated model.

#### 4.3.5. Marginal Effects

As this is a probabilistic model such as Ordinal Logit, the coefficients only allow the identification of the statistical significance of the variables, but not their direct interpretation. For this reason, marginal effects were calculated, which allow estimating how a change in each variable affects the probability of a household being in the different categories of food insecurity.

The marginal effects estimated from the Ordinal Logit model are presented in Table 7.

The results indicate that both monthly household income and the head of household’s educational level are associated with lower probabilities of severe food insecurity., especially in its most severe forms. An increase in income or educational level increases the probabilities of belonging to the food security or mild insecurity categories, while significantly decreasing the probabilities of being in situations of moderate and severe insecurity.

On the other hand, although the variable other income was not significant in the model, its interpretation remains relevant, as it was considered an important element in the study of food insecurity in the Guamote Canton. The results show that additional income has very small marginal effects on the probability of belonging to any of the food insecurity categories. This is explained because, according to the surveys conducted, the majority of households do not receive any type of additional income, which generates limited variation in this variable and reduces its explanatory capacity in the model.

Regarding the gender variable, the results show that being a woman is associated with a high probability of suffering moderate and severe food insecurity, and with a low probability of being in conditions of food security or mild insecurity, compared to men. This pattern reflects specific social and economic dynamics, such as inequality in access to resources, differences in gender roles and domestic responsibilities, which affect women more in vulnerable contexts.

This study also considered whether people lived in rural or urban areas; the results indicate that living in a rural area is associated with a higher likelihood of experiencing moderate and severe food insecurity compared to living in an urban area. This can be explained by better access to markets, basic services (such as health and education) and social assistance programs in the urban area of the Canton, in contrast to rural areas (Cebadas and Palmira), where these facilities are more limited. This access favors food acquisition and contributes to improving food security.

Finally, access to information on proper nutrition was positively associated with food insecurity, indicating a higher likelihood that households would fall into the moderate and severe food insecurity categories. Although this result may seem contradictory, it can be explained by the fact that assistance programs and nutrition education campaigns tend to target primarily the most vulnerable households. In the Guamote Canton, food insecurity is closely linked to structural factors such as poverty, low educational levels, and unstable incomes; therefore, access to information alone does not guarantee an improvement in food security. This finding suggests that nutrition education strategies must be complemented by policies aimed at strengthening households’ economic and social conditions to achieve more sustainable outcomes. Another possible explanation is reverse causality, whereby households experiencing greater food insecurity may have increased contact with nutrition education programs, community interventions, or public assistance initiatives. Therefore, the observed relationship should be interpreted cautiously and should not be considered evidence that nutritional information increases food insecurity.

## 5. Discussion

The present study examined the relationship between socioeconomic factors and food insecurity in Guamote Canton, Ecuador, highlighting household income and educational level as key of food insecurity among rural households. The findings indicate that lower income and lower educational attainment are significantly associated with higher probabilities of moderate and severe food insecurity. These results reinforce existing evidence showing that socioeconomic vulnerability remains one of the main factors associated with food insecurity in low-income rural populations [3,15,35,65,66,67].

Most surveyed households reported monthly incomes below the Ecuadorian unified minimum wage for 2024, reflecting the structural economic vulnerability affecting the population of Guamote. Limited and unstable income restricts households’ ability to access sufficient and nutritious food, increasing the risk of food insecurity [35,65,66,67]. In addition, the low proportion of households receiving complementary income sources, such as remittances or government transfers, suggests that existing social protection mechanisms remain insufficient to mitigate economic vulnerability [68,69,70,71,72].

Educational level also showed a significant negative association with food insecurity. Most heads of households had only primary or secondary education, which may limit access to stable employment opportunities, higher earnings, and nutritional knowledge. From a human capital perspective, education contributes not only to income generation but also to improved decision-making regarding food acquisition, dietary practices, and household resource management [73,74,75,76]. Similar associations between educational attainment and food security have been reported in rural populations across Latin America [40,77,78,79,80,81].

Gender differences were also evident in the analysis. Female-headed households showed a higher probability of experiencing moderate or severe food insecurity compared with male-headed households. This finding is consistent with previous literature suggesting that women in vulnerable rural contexts often face greater economic instability, unequal access to productive resources, and higher unpaid domestic workloads [22,82,83,84].

Area of residence was another significant factor associated with food insecurity. Households located in rural areas were more likely to experience food insecurity than those located in urban areas. This difference may be explained by disparities in access to employment opportunities, markets, healthcare, education, and basic infrastructure [33]. Rural households in Guamote depend largely on small-scale agriculture, which is highly vulnerable to climate variability and income instability.

An unexpected finding was the positive association between access to nutritional information and higher levels of food insecurity. However, this result may reflect the fact that food assistance and nutrition education programs are primarily targeted toward the most socioeconomically vulnerable households. Although these households may receive nutritional guidance, structural barriers such as insufficient income, unstable employment, and limited access to affordable and diverse foods may prevent them from translating nutritional knowledge into improved dietary practices [45,85].

Overall, the results show that food insecurity in the Guamote Canton is closely linked to structural socioeconomic inequalities, particularly factors such as income and educational attainment. These findings provide relevant evidence for the design of public policies aimed at strengthening households’ economic capacities, expanding educational opportunities, improving social protection systems, and optimizing the allocation of resources for vulnerable populations. The implementation of comprehensive interventions in these areas would help improve food security and reduce nutritional vulnerability, both in Guamote and in other regions with similar socioeconomic characteristics.

### Limitations

This study has several limitations that should be taken into account. First, its cross-sectional design does not allow for the establishment of causal relationships between socioeconomic factors and food insecurity. Second, the data on household income were based on information provided by the respondents themselves and may therefore be subject to measurement errors or reporting biases. Third, factors such as cooking skills, nutritional knowledge, eating habits, knowledge of food preparation, and access to social support programs—which could influence the results regarding food insecurity—were not measured. Additionally, the proportional odds (parallel lines) assumption of the Ordinal Logit model was evaluated and showed evidence of violation. Therefore, the estimated associations should be interpreted with caution, as the effects of some explanatory variables may differ across the levels of food insecurity. Finally, the results are specific to the Canton of Guamote and should be interpreted with caution when generalized to other regions of Ecuador.

## 6. Conclusions

The present study found significant associations between household income, educational level, and food insecurity, which answers the research question. The results obtained from the application of the Ordinal Logit model indicate that higher household income and education levels of the head of the household are associated with lower probabilities of food insecurity among households in Guamote Canton, providing empirical support for the proposed hypothesis regarding the relationship between these variables.

Likewise, it has been identified that households in this Canton have low incomes and an educational level that, for the most part, does not exceed secondary education, placing them in a context characterized by socioeconomic limitations. Similarly, it was found that only a small proportion of households experience food security, while food insecurity remains prevalent and is associated with various socioeconomic conditions related to access to nutritious food.

Finally, the use of the Ordinal Logit model proved appropriate for analyzing food insecurity, allowing for the interpretation of marginal effects through quantification and the examination of statistical relationships according to their signs. In this way, this study contributes to a better understanding of the associations between food insecurity, income levels, educational attainment, and other socioeconomic factors. Thus, the findings provide evidence on the current context of food insecurity in Guamote Canton and may serve as a basis for the design of strategies and policies aimed at improving access to food and household well-being.

## Figures and Tables

**Table 1:** Number of surveys per parish.

Parish	Residents	Ratio	Surveys
Matriz	19,382	0.54	199
Cebadas	6414	0.18	66
Palmira	9973	0.28	103
Total	35,769	1.00	368

Note. The table presents the proportional allocation of the sample across the three parishes of Guamote Canton.

**Table 2:** Coding the Independent Variables.

Variable	Coding
Household income	Family income per month. Less than $460 = 1 Between $460–$920 = 2 Between $921–$1381 = 3 Between $1382 or more = 4 Other income. Bonds: less than or equal to $50 = 1; Greater than $50 = 2 Internal remittances: less than or equal to $100 = 3; over $100 = 4 International remittances: less than or equal to $200 = 5; greater than $200 = 6. None = 7
Education level of the head of household	Level of education of the head of the household. None = 1 Primary = 2 Secondary = 3 Superior = 4 Postgraduate = 5 Other = 6
Gender	Male = 1 Female = 2
Area of residence	Urban = 1 Rural = 2
Household size	How many people live in your household, including you? Less than or equal to 3 = 1 4 to 7 = 2 Equal to or greater than 8 = 3
Access to nutrition information	Do you have information about adequate nutrition in your home? Yes = 1 No = 2
Ethnicity	What is their ethnicity? Indigenous = 1 Afro-Ecuadorian = 2 Mestiza = 3 Mulatto = 4 White = 5 Montubia = 6 Other = 7
Type of residence	Where do you live? Own = 1 Rented = 2 Courtesy of family/friends = 3 In antichresis = 4 Other = 5

Note. The table presents the coding scheme used for the independent variables included in the Ordinal Logit model.

**Table 3:** Regression of the models.

	Ordinal Logit		Ordinal Probit	
Food Insecurity	Coefficient	*p*-Value	Coefficient	*p*-Value
Household income	−0.6392	0.001	−0.3677	0.001
Other income	−0.0365	0.373	−0.0209	0.392
Education level of the head of household	−0.3664	0.008	−0.2249	0.005
Gender	0.4703	0.033	0.2712	0.032
Area of residence	−0.4635	0.030	−0.2611	0.034
Household size	0.1261	0.497	0.0571	0.596
Access to nutrition information	0.4666	0.033	0.2657	0.034
Ethnicity	−0.1202	0.390	−0.0720	0.375
Place of residence	0.0079	0.960	0.0158	0.861

Note. The table presents the estimated coefficients and *p*-values for the ordinal Probit and Ordinal Logit models. In the table, it is observed that all variable coefficients in the Ordinal Probit model are smaller in magnitude compared to the Ordinal Logit model. This is because the Probit model uses a cumulative normal distribution, while the Logit uses a cumulative logistic function. Although these scales are different, the changes in terms of direction and relative interpretation are consistent between both models. However, this difference in magnitudes does not affect the general conclusions, as the signs of the coefficients and the significant variables remain similar in both cases.

**Table 4:** Criteria for model selection.

Model	Chi-Square Value	Pseudo R Value	Maximum Likelihood	AIC	BIC
Ordinal Logit	61.44	0.0654	−439.06898	902.138	949.035
Ordinal Probit	59.65	0.0635	−439.96299	903.926	950.823

Note. AIC: Akaike Information Criterion; BIC: Bayesian Information Criterion. Lower AIC and BIC values indicate better model fit. *p*-values correspond to the likelihood ratio test. Own elaboration.

**Table 5:** Correlation matrix of independent variables.

Variable	1	2	3	4	5	6	7	8	9
Household income	1.000								
Other income	0.1382	1.000							
Education level of head of household	0.4000	0.2088	1.000						
Gender	0.0810	0.0399	−0.0911	1.000					
Area	−0.2290	−0.0922	−0.2747	0.1134	1.000				
Household size	0.0582	−0.0034	−0.1456	0.0064	−0.0770	1.000			
Access to nutrition information	−0.2328	−0.1072	−0.4175	0.0355	0.0427	0.0647	1.000		
Ethnicity	0.2837	0.1621	0.2842	0.0479	−0.2488	0.0415	−0.1308	1.000	
Type of residence	−0.0190	−0.0389	0.1390	−0.1110	−0.1049	−0.0851	−0.0039	−0.0271	1.000

Note. Pearson correlation coefficients below 0.80 suggest no evidence of problematic multicollinearity. Own elaboration.

**Table 6:** Model Regression.

Inseguridad Alimentaria	Odds Ratios (OR)	Coefficient	*p*-Value	Confidence Intervals 95% OR
Household income	0.5277	−0.6392	0.001 ***	0.3641–0.7649
Other income	0.9642	−0.0365	0.373	0.8899–1.0447
Education level of head of household	0.6931	−0.3665	0.008 ***	0.5289–0.9086
Gender	1.6004	0.4703	0.033 **	1.0396–2.4639
Area	0.6291	−0.4635	0.030 **	0.4137–0.9565
Household size	1.1344	0.1261	0.497	0.7882–1.6327
Access to nutrition information	1.5945	0.4666	0.033 **	1.0374–2.4508
Ethnicity	0.8868	−0.1202	0.390	0.6744–1.1660
Place of residence	1.0079	0.0079	0.960	0.7428–1.3676
Chi-square value	61.44			
Average VIF value	1.19			

Note. OR = Odds Ratio; CI = Confidence Interval; VIF = Variance Inflation Factor. *p*-values correspond to the z-statistic. Statistical significance: *** *p* < 0.01; ** *p* < 0.05. Own elaboration.

**Table 7:** Marginal Effects in Partial Derivatives.

	Household Income	Education Level of the Head of Household	Other Income	Gender	Area	Home Information
Prediction	dy/dx	dy/dx	dy/dx	dy/dx	dy/dx	dy/dx
Food Security	0.0524879 ↑	0.0300928 ↑	0.0029936 ↑	−0.0386209 ↓	0.038059 ↑	−0.0383136 ↓
Mild Food Insecurity	0.0869371 ↑	0.0498435 ↑	0.0049583 ↑	−0.0639688 ↓	0.0630381↑	−0.0634599↓
Moderate Food Insecurity	−0.0498539 ↓	−0.0285827 ↓	−0.0028433 ↓	0.0366828 ↑	−0.0361491 ↓	0.036391 ↑
Severe Food Insecurity	−0.0895711 ↓	−0.0513536 ↓	−0.0051085 ↓	0.0659069 ↑	−0.064948 ↓	0.0653826↑

Note. Marginal effects (dy/dx) indicate the change in predicted probability associated with a one-unit increase in the explanatory variable while holding other variables constant. HH = Head of Household, ↑ indicates an increase in the probability, ↓ indicates a decrease in the probability. Own elaboration.

## Data Availability

The original contributions presented in this study are included in the article. Further inquiries can be directed to the corresponding author.

## Appendix A

**Table A1:** Questions included in the ELCSA for households with no children under 18.

Questions
Q1. In the past 3 months, due to a lack of money or other resources, have you ever worried that your household would run out of food?
Q2. In the past 3 months, due to a lack of money or other resources, has your household ever run out of food?
Q3. In the past 3 months, due to a lack of money or other resources, did your household ever stop eating a healthy diet?
Q4. In the past 3 months, due to a lack of money or other resources, did you or any adult in your household ever eat a diet consisting of a limited variety of foods?
Q5. In the past 3 months, due to a lack of money or other resources, did you or any adult in your household ever skip breakfast, lunch, or dinner?
Q6. In the past 3 months, due to a lack of money or other resources, did you or any adult in your household ever eat less than you should have?
Q7. In the past 3 months, due to a lack of money or other resources, did you or any adult in your household ever feel hungry but not eat?
Q8. In the past 3 months, due to a lack of money or other resources, did you or any adult in your household ever eat only once a day or go without eating for an entire day?

Note. The table shows the main questions used to measure food insecurity. Source: Prepared by the author based on information provided by the ELCSA.
